# Autoproteolytic Activation of Bacterial Toxins 

**DOI:** 10.3390/toxins2050963

**Published:** 2010-05-06

**Authors:** Aimee Shen

**Affiliations:** Department of Pathology, Stanford School of Medicine, 300 Pasteur Drive, Stanford, California 94305, USA; Email: ashen2@stanford.edu; Tel.: +1-650-736-4099; Fax: +1-650-725-7424

**Keywords:** cysteine protease domain (CPD), MARTX toxin, glucosylating toxin (GT), inositol hexakisphosphate (InsP_6_), glucosyltransferase (Glc), structure activity relationship (SAR)

## Abstract

Protease domains within toxins typically act as the primary effector domain within target cells. By contrast, the primary function of the cysteine protease domain (CPD) in Multifunctional Autoprocessing RTX-like (MARTX) and *Clostridium* sp. glucosylating toxin families is to proteolytically cleave the toxin and release its cognate effector domains. The CPD becomes activated upon binding to the eukaryotic-specific small molecule, inositol hexakisphosphate (InsP_6_), which is found abundantly in the eukaryotic cytosol. This property allows the CPD to spatially and temporally regulate toxin activation, making it a prime candidate for developing anti-toxin therapeutics. In this review, we summarize recent findings related to defining the regulation of toxin function by the CPD and the development of inhibitors to prevent CPD-mediated activation of bacterial toxins.

## 1. Autoproteolytic Activation of Bacterial Toxins

**Figure 1:**
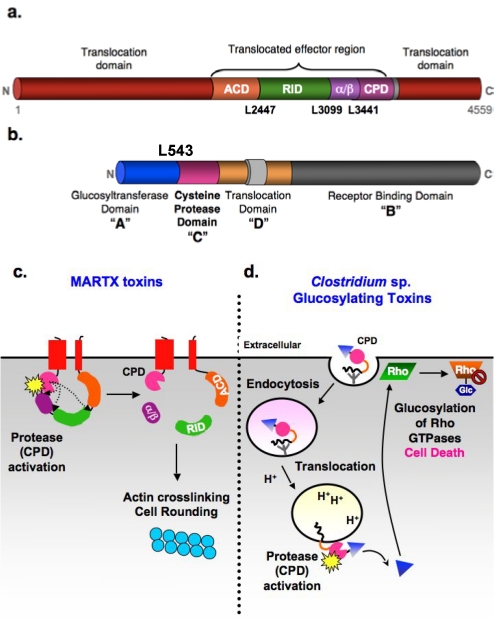
Autoproteolytic activation of MARTX and glucosylating toxin families by the CPD. ** (a)** Domain organization of *V. cholerae* MARTX toxin. Conserved glycine-rich repeat regions in the *N*- and *C*-termini of MARTX toxins (MARTX conserved, red); actin crosslinking domain (ACD, orange); Rho-inactivating domain (RID, green); α/β hydrolase domain (α/β, purple), cysteine protease domain (CPD, pink). Cleavage sites are shown. **(b) **ABCD domain organization of *C. difficile* glucosylating toxins. A: Glucosyltransferase (Glc) “activity” domain; B: receptor “binding” domain; C: cysteine protease “cutting” domain; and D: hydrophobic translocation “delivery” domain. Cleavage site in TcdB is marked **(c)** Schematic model for CPD-mediated activation of *V. cholerae* MARTX toxin. Upon encountering a eukaryotic cell, the *N*- and *C*-terminal MARTX_Vc_ conserved regions insert into the plasma membrane and form a pore that permits translocation of the toxin central region across the cell membrane. Following toxin entry, the CPD binds inositol hexakisphosphate (InsP_6_, starburst), resulting in activation of its protease activity. The CPD first cleaves at Leu3441, the preferred autoprocessing site, then Leu2447, followed by Leu3099 (least preferred cleavage site) [9]. Processing of MARTX_Vc_ by the CPD releases the RID and (α/β) domains into the cytosol, while the ACD and CPD remain tethered to the membrane. Cleavage of MARTX_Vc_, particularly at Leu2447, activates the actin crosslinking activity of the ACD. **(d)** Schematic model for CPD-mediated activation of *Clostridium* sp. glucosylating toxins. Binding of the toxins to unidentified receptors results in their uptake by receptor-mediated endocytosis. Acidification of the early endosome triggers toxin translocation of the Glc domain (triangle). This is the only part of the protein released into the cytosol, presumably due to CPD-mediated autoproteolysis [13]. Whether the CPD itself is translocated, and when and where it is activated by InsP_6_ (starburst) is unknown, although it likely occurs on the cytosolic face of the endosomal membrane.

Many bacterial toxins whose targets are within host cells require proteolytic activation for their function. Whereas most toxins, such as cholera toxin and anthrax toxin, are cleaved by a eukaryotic cell protease, a subset of bacterial toxins is autoproteolytically activated by an internal cysteine protease domain (CPD). The CPD is found within two multidomain toxin families: the Multifunctional Autoprocessing RTX-like toxins (MARTX) toxins and the *Clostridium* sp. glucosylating toxins (GTs) (**Figure 1a and b**). Because the CPD is directly activated by the eukaryotic-specific small molecule inositol hexakisphosphate (InsP_6_), the CPD acts as a biosensor that appears to allow for temporal and spatial regulation of toxin activation. Once toxins translocate across eukaryotic cell membranes, the CPD binds to InsP_6_ within the cell (K_D_ ~ 1-2 µM) [3,4,5] and induces toxin autoprocessing. Cleavage of the toxins by the CPD releases cognate effector domains and subsequently enhances toxin function [6,7,8,9]. Importantly, InsP_6_ is found at concentrations between 5-100 µM within the cytosol of mammalian cells, exhibits a long half-life in cells, and may be localized to cellular membranes and the nucleus [10,11,12].

### 1.1. MARTX toxins

The MARTX toxins are a newly discovered family of toxins that are encoded in the genomes of a number of Gram-negative bacteria [14]. Although only a few toxin members have been studied to date, MARTX toxins have been shown to modulate the virulence of *Vibrio cholerae*, *Vibrio vulnificus*, and *Vibrio anguillarum*. The MARTX toxin of *V. cholerae*, the causative agent of cholera, is produced by nearly all clinical and environmental isolates of *V. cholerae* [15,16,17]. Although it is not cytotoxic, it induces cell rounding and enhances colonization in a mouse model of infection [18,19,20,21]. By contrast, MARTX toxins of the marine pathogens *V. vulnificus* and *V. anguillarum* are cytotoxic and function as key virulence factors [22,23,24]. 

MARTX toxins are some of the largest bacterial proteins identified to date, with molecular weights often in excess of 450 kDa [14]. They are characterized by conserved *N*- and *C*-terminal repeat regions with similarity to RTX toxins that flank a central effector domain region (**Figure 1a**). These repeat regions have been proposed to form a pore that allows the central effector region to autotranslocate across host cell membranes [18]. While the composition of the central region varies across MARTX toxin family members, the most *C*-terminal effector of MARTX toxins is always the InsP_6_-sensing cysteine protease domain (CPD). In *V. cholerae* MARTX toxin, the best characterized family member, the CPD cleaves the toxin at multiple sites to release discrete effector domains (**Figure 1c**) [9,25].

Aside from the CPD, the activities of most MARTX effector domains remain uncharacterized. The functions of only two other effector domain functions have been identified to date; both these domains are found within *V. cholerae* MARTX toxins and disrupt host cell actin dynamics. The actin crosslinking domain (ACD) has homology to ATP-dependent ligases and covalently crosslinks actin monomers together through an atypical glutamate-lysine crosslink [26,27,28,29]. As a result, the ACD prevents growth of actin filaments and depletes the monomeric actin pool, which leads to cell rounding due to destruction of the actin cytoskeleton. The Rho Inactivating Domain (RID) domain inhibits small Rho GTPase activity through an as yet undefined mechanism [30]. 

### 1.2. *Clostridium sp.* glucosylating toxins

Similar to MARTX toxins, *Clostridium* sp. glucosylating toxins are multidomain toxins that alter target cell cytoskeletal dynamics in a manner dependent on CPD-mediated toxin autoprocessing (**Figure 1b and d**). Glucosylating toxins are large (~250 kDa) proteins with an ABCD toxin structure (A, biological activity; B, binding; C, cutting; D, delivery) [7,31,32]. The “biological activity” (A) is conferred by the *N*-terminal glucosyltransferase (Glc) domain, which glucosylates Rho GTPases (such as RhoA and Ras) at a conserved Thr residue [33,34]. This covalent modification prevents Rho GTP-GDP exchange and irreversibly inhibits Rho GTPase function, leading to disruption of epithelial barrier junctions, inhibition of Rho-mediated signaling, and ultimately cell death and inflammation [35]. The “binding activity” (B) of GTs is mediated by the *C*-terminal repeat region, which forms a solenoid structure that probably binds sugar moieties on cell surface receptors (the identity of which has not been determined). The toxins are then taken up by receptor-mediated endocytosis. 

As “short-trip” toxins, acidification of the early endosomal compartment induces conformational changes in GTs that results in membrane insertion and toxin translocation. The central, hydrophobic region is thought to confer this “delivery activity” (D) [36]. While the mechanism by which this occurs remains unclear, the central region likely mediates translocation of the Glc domain across the endosomal membrane into the target cell cytosol. The CPD domain presumably is also transferred, but this has not been directly tested. Nevertheless, binding of InsP_6_ to the CPD (either in the endosome or in the host cytosol) results in CPD activation and cleavage of GTs at the Glc domain-CPD junction [3,37,38]. This “cutting” (C) event releases the Glc domain from the endosome, an event that presumably improves access of the Glc domain to its Rho GTPase substrates at the membrane.

The glucosylating toxins (GTs) are produced by select *Clostridium* sp. pathogens, which are Gram-positive, anaerobic, spore-forming bacteria. Members of the GT family include TcdL and TcdH of *C. sordellii*, TcnA of *C. novyi*, and *C. perfringens* types B and C, but the prototypical members are TcdA and TcdB of *C. difficile*. This latter bacterium is of particular interest as it is the leading cause of nosocomial diarrhea worldwide and is associated with significant morbidity and cost in health care-associated settings [39,40]. The glucosylating toxins TcdA and TcdB are the primary causes of *C. difficile* infection (CDI), with TcdB being the more active of the two toxins and essential for virulence [41]. The relevance of these toxins to disease is underscored by the observation that a strain that hyper-produces TcdA and TcdB causes more frequent and severe cases of CDIs [40,42]. Thus, the development of inhibitors that can inhibit TcdA and TcdB function or activation should have considerable therapeutic utility. 

## 2. Autoprocessing Cysteine Protease Domains

**Figure 2:**
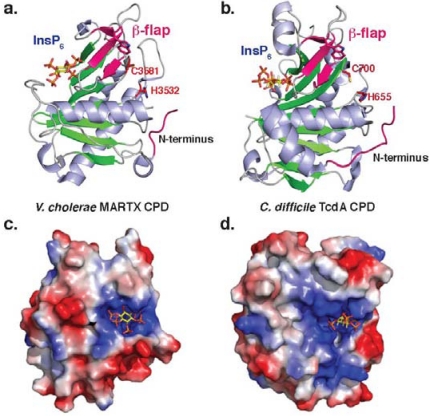
**Structure of bacterial CPDs.** Ribbon structure of **(a)***V. cholerae* MARTX CPD (PDB ID: 3EEB) and **(b)***C. difficile* TcdA CPD (PDB ID: 3HO6). Electrostatic surface potential of **(c)***V. cholerae* MARTX CPD and (**d**) *C. difficile* TcdB CPD as viewed from above the InsP_6_ binding site. Blue denotes positively charged surface; red denotes negatively charged surface. InsP_6_ is shown in the binding site as a stick model. Note that the orientation of the InsP_6_ molecule differs between the two structures.

Although MARTX and GT toxin families differ in their activities and domain organization, they share a common cysteine protease domain (CPD) that proteolytically processes toxin family members. Despite differing in 60% of their amino acid residues, both MARTX and GT CPDs have a similar overall fold that resembles a canonical caspase-like fold **(Figure 2**a and b). As clan CD proteases (MEROPS classification) [43], both bacterial CPDs and caspases have a central β-sheet that is surrounded by α-helices [44]. Unlike the caspases, however, bacterial CPDs contain an InsP_6_ binding site and a region termed the β-flap (described below). Structures of both MARTX and TcdB CPDs reveal that the InsP_6_ binding site is comprised of numerous positively charged residues that trap a single negatively-charged InsP_6_ molecule [4,25,45]. Interestingly, the mechanism of InsP_6_ binding differs slightly between Tcd and MARTX CPDs, with InsP_6_ lying flat on the surface of the protease in *V. cholerae* MARTX CPD, and InsP_6_ being wedged into the side of TcdA CPD **(Figure 2**c and d) [45]. Both proteases, however, have similar low micromolar affinities for InsP_6_[4,5,37].

Notably, the InsP_6_ binding site is physically distinct from the active site, indicating that InsP_6_ likely functions as an allosteric activator of bacterial CPDs. Although a number of studies have shown that InsP_6_ binding improves access of the CPD to its substrates and inhibitors [3,4,25], the mechanism by which InsP_6_ binding is communicated to the active site has not been fully characterized. Current work suggests that the β-flap region - the second structural feature in which bacterial CPDs differ from other clan CD proteases - transduces the InsP_6_ binding to the active site. Mutation of key residues in the β-flap disrupt CPD function [4,5,37,45], and residues in this region have been shown to be resistant to limited proteolysis in the presence of InsP_6 _[25]. This latter result suggests that InsP_6_ binding induces stabilization of the β-flap in the activated protease. Interestingly, the β-flap region of TcdA CPD is larger than that of *V. cholerae* CPD, with TcdA CPD containing an additional alpha-helix [45]; the functional significance of this addition is unknown. 

**Figure 3:**
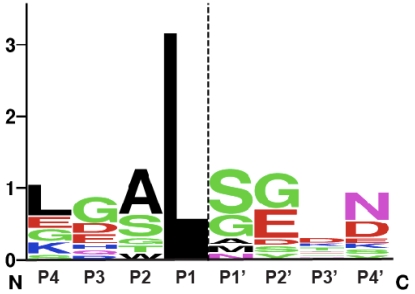
**Sequence logo representation of MARTX and GT CPD consensus cleavage site.** The sequence logo was created using natural MARTX CPD cleavage sites mapped by intact mass spectrometry [9] and cleavage sites mapped and/or inferred in GT CPDs [38,49], see http://weblogo.berkeley.edu. The dashed line indicates the scissile bond.

This mechanism of allosteric regulation by a small metabolite is unique among proteases. Nevertheless, bacterial CPDs exhibit many similarities in substrate recognition to clan CD proteases. In particular, like the caspases, the substrate specificity of MARTX CPDs is governed by the P1 residue, the amino acid immediately *N*-terminal to the scissile bond [9,25,46,47,48]. Just as caspases cleave almost exclusively after P1 Asp residues, bacterial CPDs appear to cleave exclusively after P1 Leu residues: all known MARTX and GT toxin autoprocessing sites occur after Leu residues [3,9,25,49]. In addition, like the caspases, MARTX CPDs appear to exhibit a preference for a small amino acid in the P1’ position, the residue immediately *C*-terminal to the scissile bond. A comparison of known and inferred cleavage sites within MARTX and GT toxins suggests that small residues in the P1’ position are favored (Figure 3). Furthermore, the CPD has been shown to cleave more efficiently when the P1’ residue is an Ala *versus* a Ser residue, while the presence of a bulky P1’ Leu residue is sufficient to abrogate CPD-mediated processing [9]. 

## 3. Development of Inhibitors of Bacterial CPD Protease Activity

The restraints on substrate specificity determined by cleavage site comparisons are also observed in studies with MARTX CPD inhibitors [9]. Inhibitors of *V. cholerae* MARTX CPD processing were recently identified in a screen of a focused library of cysteine protease inhibitors. All CPD inhibitors derived from the screen contain a Leu in the P1 position, the residue immediately *N*-terminal to the scissile bond. They were also all functionalized with an aza-epoxide group, which reacts with the caspases [50]. The screen produced a small structure-activity relationship (SAR) series that shows remarkable similarities to an SAR series previously performed on the caspases. In particular, the regio- and stereochemistry at the epoxide moiety influences inhibitor potency for both *V. cholerae* CPD and caspase-3, with *S,S* > *trans* > *R,R* [9,51]. 

**Figure 4:**
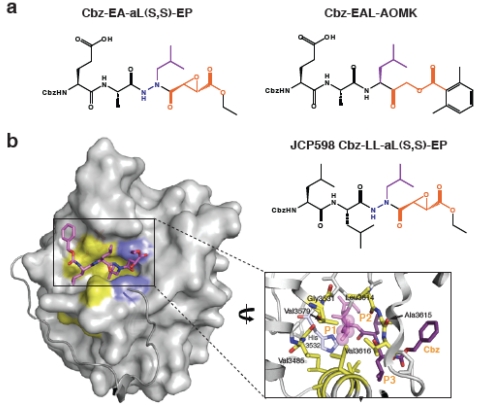
**Chemical inhibition of MARTX CPD activity.** (**a**) Structures of the most potent MARTX CPD inhibitors. Navy blue indicates aza-linkage; purple highlights P1 Leu, and orange denotes electrophile warhead. **(b) **Surface topology of activated MARTX_Vc_ CPD bound to an aza-peptide epoxide inhibitor. Hydrophobic residues in the substrate binding cleft are in yellow. The aza-peptide epoxide inhibitor (JCP598) is shown as a stick model bound in the substrate binding pocket. The *N*-terminus is shown as a grey ribbon, terminating at Ile3437 and highlighting the threading of this region along the surface of the core domain.

The crystal structure of *V. cholerae* CPD bound to the most potent inhibitor from the screen (JCP598, Z-LL-azaL-EP, **Figure 4a**) provided the most convincing evidence that MARTX CPDs and caspases share similar mechanisms of substrate recognition and catalysis [9]. In both the *V. cholerae* CPD and caspase-3 structures bound to aza-epoxide inhibitors, the catalytic Cys of *V. cholerae* CPD attacks on the C3 position of the expoxide rather than the C2. Furthermore, the catalytic residues of *V. cholerae* MARTX CPD and caspase-3 exhibit similar geometries around the P1-P1’ peptide bond, and the S1 binding pockets (that recognize the P1 residue) are similarly aligned. Indeed, the S1 pocket of *V. cholerae* MARTX CPD forms a deep, hydrophobic cavity that perfectly accommodates a Leu residue (**Figure 4b**), and residues that form this S1 pocket are conserved across MARTX and GT CPDs. Notably, the structure of aza-epoxide inhibited *V. cholerae* MARTX CPD is essentially superimposable with the recently solved structure of the uncleaved form of the protease. This uncleaved form carries the native P1 Leu and non-native TEV protease cleavage site [25]. The P1 Leu residues of both MARTX CPD structures are similarly aligned, while mutational analyses of the P2 site also support the conclusion that the primary substrate specificity determinant is a P1 Leu [25]. 

As observed with the natural cleavage substrates of MARTX toxins, the P2 and P3 positions of the inhibitors do not strongly contribute to substrate recognition [9]. For example, no difference in inhibitor potency was observed between a Cbz-Leu-Leu-azaLeu (Z-LLaL-EP) epoxide inhibitor and Cbz-Asp-Ala-azaLeu (Z-EAaL-EP) epoxide inhibitor. Furthermore, the S2 and S3 sub-sites in the crystal structure of inhibitor-bound MARTX CPD do not make contacts with the P2 and P3 side chains of the inhibitor; rather, the protease interacts with the peptide backbone of the inhibitor. These conclusions are consistent with SAR series results, indicating that inhibitors carrying P2 and P3 residues were more potent than inhibitors with only a single P1 Leu. 

The functional group of the P1 Leu inhibitors also affects inhibitor potency. Whereas inhibitors with a P1 Leu and acyloxymethylketone (AOMK) functional group, which reacts with caspases [52], block the activity of *V. cholerae* MARTX CPD, inhibitors with the same peptide sequence as JCP598 (but different reactive groups) do not inhibit CPD function [9]. Specifically, MG132, a common proteasome inhibitor (Cbz-LLL-aldehyde) and Z-L_3_VS (Cbz-LLL-vinyl sulfone) do not block *V. cholerae* CPD autocleavage. The lack of inhibitory activity of Z-L_3_VS may reflect the preference of MARTX CPDs for small P1’ residues in the consensus cleavage site sequence. Indeed, the S1’ subsite (which interacts with the P1’ residue) is flat and featureless and may not accommodate large constituents in the P1’ position. Nevertheless, making contacts with the S1’ subsite would appear to be important for inhibitor potency given the lack on inhibitor activity of MG132.

Although the aza-epoxide inhibitors identified in the screen are designed to covalently inhibit cysteine proteases, they failed to irreversibly react with *V. cholerae* MARTX CPD under standard assay conditions (A. Shen, unpublished data). The apparent slow reactivity of *V. cholerae* MARTX CPD with the aza-epoxide inhibitors may reflect the rapid rate with which InsP_6_ likely binds to and dissociates from the CPD relative to inhibitor binding. In contrast, when the aza-epoxide inhibitor was co-crystallized with *V. cholerae* CPD in the presence of InsP_6_, the inhibitor likely had sufficient time to react with the enzyme constrained in the crystal lattice. The lack of reactivity of the aza-epoxide inhibitors may also reflect the need for optimization of substrate binding or electrophile reactivity to *V. cholerae* CPD. To the latter point, functionalized alkylating agents have been shown to react with the sole catalytic Cys of *V. cholerae* CPD in a manner that is enhanced by the presence of InsP_6_[9,25].

Nevertheless, MARTX CPD inhibitors are capable of reducing CPD-mediated processing of MARTX toxin during growth of *V. cholerae* in broth culture [9]. While the inhibitors are not effective at preventing CPD autocleavage in the native toxin, they do prevent processing of the toxin by the CPD at distal sites. Although they have limited potency on the native toxin, the CPD inhibitors were useful in determining the relative affinity of the CPD for its different cleavage sites, which correlated the relative affinities determined using *in vitro* cleavage reactions. Notably, the inhibitors were also capable of preventing MARTX toxin activity (specifically actin crosslinking activity) during intoxication of target cells. The aza-epoxide inhibitors were the most potent, since they were more cell permeable than the AOMK inhibitors; Cbz-LLaL-EP was the most cell permeable inhibitor. While the specificity of these inhibitors in complex mixtures still needs to be assessed, these results strongly suggest that CPD inhibitors can prevent the autoproteolytic activation of MARTX toxins inside target cells. Thus, the MARTX CPD inhibitors provide a starting point for the development of active site inhibitors of MARTX CPDs and potentially GT CPDs (given that S1 residues in MARTX and GT CPDs appear to be conserved).

## 4. Requirement of CPD-Mediated Processing for Toxin Activity

Although CPD-mediated processing of *V. cholerae* MARTX and *C. difficile* Tcd toxins is necessary for optimal toxin function, it is not absolutely required. Toxins carrying mutations in the catalytic Cys of both *V. cholerae* MARTX and *C. difficile* TcdB CPDs can still exert cytopathic effects on cells, but the toxin activity is significantly decreased [3,4,6,8]. Why proteolytic cleavage enhances effector domain function has not been characterized. The known substrates of both *V. cholerae* MARTX and Tcd toxins are often localized to the host cell plasma membrane, so it has been hypothesized that CPD-mediated processing improves access of toxin effector domains to their substrates. 

*C. difficile* glucosylating toxins are cleaved by the CPD after a single conserved Leu at the glucosyltransferase (Glc) domain-CPD junction [3,49]. This cleavage event releases the Glc domain from the endosomal membrane into the cytosol, while the remainder of the toxin remains membrane-associated (**Figure 1b**, [13,49]). This cleavage event likely enhances access of the Glc domain to its Rho GTPase substrates at the plasma membrane given that a membrane localization domain (MLD) was recently identified in its *N*-terminus [53]. This MLD was recently shown to be sufficient to mediate plasma membrane localization, with enrichment observed at cell-cell junctions in particular. An analysis of the localization of the Glc domain and its activity in toxins carrying mutations of the CPD cleavage site and/or CPD catalytic residues will be important for determining the precise function of the CPD in regulating *Clostridium* sp. glucosylating toxin activation. Such analyses will be greatly facilitated with the advent of new tools for manipulating *C. difficile* genetically [54]. 

In contrast with the glucosylating toxins, the precise mechanism by which CPD-mediated processing increases effector domain activity in MARTX toxins is less apparent. Whereas the CPD cleaves *Clostridium* sp. glucosylating toxins at a single site, the CPD processes MARTX toxins at multiple sites. The cleavage sites all occur after a Leu residue and are found at interdomain boundaries predicted to be disordered [9]. The identity of cleavage sites was confirmed by mutational analyses with both recombinant and native MARTX toxin. These analyses indicate that these three sites are the primary cleavage sites in the toxin, although a small amount of second site processing of these toxins can be observed in a mutant in which all three P1 Leu cleavage sites have been mutated to Ala residues [9]. It should be noted that a fourth cleavage site was mapped by Prochazkova *et al.* [25] using *N*-terminal sequencing of *in vitro* cleavage reactions of recombinant *V. cholerae* MARTX toxin. However, extensive Western blot analyses of wildtype and cleavage site mutants indicate that this site is not physiologically relevant in the native toxin [9].

CPD-mediated processing separates the actin crosslinking domain (ACD), Rho-inactivating domain (RID), and α/β hydrolase domains (**Figure 1a**). While these cleavage events should liberate the RID and α/β hydrolase domains from the membrane, both the ACD and CPDs are predicted to remain tethered to the membrane. The membrane localization of these domains may be important to their function, perhaps by optimizing access of the ACD and CPD to their substrates. Indeed, the CPD is always found adjacent to the *C*-terminal MARTX conserved region, while the ACD in other MARTX toxins is always found just downstream of the *N*-terminal MARTX conserved region. Tethering of the CPD to the membrane likely facilitates processing of the toxin at distal sites, by keeping the substrates in close proximity. The dependence of the ACD domain on CPD-mediated processing remains less clear. Studies of *V. cholerae* cleavage site mutants indicate that processing of the ACD-RID junction is necessary for optimal ACD activity [9]. Nevertheless, cleavage at any site would appear to stimulate ACD activity, although why this is observed is unknown. Further studies of the ACD and its interaction with its substrate will likely provide important insight into the functional requirements of this domain and the role of CPD-mediated processing in stimulating its activity.

The dependence of the remaining *V. cholerae* effector domains on CPD-mediated processing has not yet been tested. Further analysis of the biochemical functions of the RID and α/β hydrolase domains will likely provide insight into any role that the CPD may play in activating the function of these effector domains. These studies will require that a function for the α/β hydrolase domain be determined, since there is no information on the activity or localization of this domain. Likewise, a more thorough characterization of the function of the RID domain would contribute to an understanding of the role of proteolysis in activating RID function. Although the RID domain has been shown to disrupt Rho GTPase function [30], the mechanism by which this occurs is unknown. CPD-mediated processing should liberate the RID domain from MARTX toxin at the membrane. However, a recent study suggests that the RID should remain associated with the membrane through its *N*-terminal MLD domain [53], which preferentially localizes to cell-cell junctions and is necessary for optimal RID activity. If these sites are where the putative RID substrate is enriched, CPD-mediated cleavage may similarly enhance access of the RID domain to its substrates. Indeed, localization of the RID to the membrane has been shown to significantly increase its ability to induce cell rounding [53]. Alternatively, CPD-mediated processing may function to produce free *N*- and *C*-termini that are necessary for optimal RID enzymatic activity.

## 5. Conclusions and Perspectives

Analyses of *V. cholerae* MARTX toxin will likely be transferable to other MARTX toxins, which typically contain RID, ACD, and/or α/β domains. Studies of other MARTX toxins will be greatly enhanced by mapping CPD cleavage sites, since these will define effector domain boundaries and contribute to a more global understanding of the role of this protease domain in toxin regulation. In general, the CPD appears to function analogously to viral polyprotein processing proteases such as those produced by picornaviruses (such as poliovirus and Hepatitis C). Viruses often use a conserved internal protease domain to proteolytically process the single polyprotein precursor into discrete functional domains that mediate viral replication and assembly [55]. Notably, both viral and CPD autoprocessing proteases exhibit a high degree of sequence specificity but poor transcleavage activity [56]. As a result, these proteases do not appear to cleave substrates in their target host cells. Consistent with this proposal, a MARTX toxin that carries only the CPD in the central translocated region does not overtly affect the morphology of target cells exposed to this mutant toxin (A. Shen, unpublished data), strongly suggesting that the CPD does not have many substrates within target cells aside from the MARTX toxins. 

Nevertheless, just as viral polyprotein processing enzymes have been prime targets for therapeutic intervention [57], the CPDs of both MARTX and *Clostridium* sp. glucosylating toxins are excellent targets for drug design. The *C. difficile* glucosylating toxin CPDs are particularly good candidates because TcdA and TcdB toxins are the primary factors responsible for *C. difficile*-associated disease [40]. Given that *C. difficile* infections are often promoted by antibiotic administration, targeting virulence rather than viability will likely be the most effective strategy for controlling *C. difficile*-associated disease. Likewise, given that MARTX toxins are comprised of multiple effector domains that vary among toxin family members [14], targeting the conserved CPD domain will likely be a more effective strategy at inhibiting MARTX toxin activation that developing inhibitors against each domain. MARTX CPD inhibitors would have particular applicability in aquaculture settings where infection by *V. anguillarum* and *V. vulnificus* infection of fish and bivalves is particularly costly to the seafood industry.

The identification of the first active site inhibitors of bacterial CPDs has provided an excellent starting point for the optimization of inhibitors with greater potency, selectivity, and stability, and the structure of inhibitor-bound CPD may serve as a platform for inhibitor modeling. An alternative strategy to targeting the active sites of these proteases may be to prevent the activation of bacterial CPDs by restraining their conformational mobility and thus allosteric activation [58]. Thus, a better understanding of the mechanism of CPD activation will likely lead to more effective strategies for preventing toxin activation.
